# The role of design engineers: Evidence from intra-firm knowledge and collaboration networks

**DOI:** 10.1371/journal.pone.0298089

**Published:** 2024-02-23

**Authors:** Jisoo Hur, Junseok Hwang, Keungoui Kim

**Affiliations:** 1 Technology Management, Economics and Policy Program, College of Engineering, Integrated Major in Smart City Global Convergence, Seoul National University, Seoul, Republic of Korea; 2 School of Applied Artificial Intelligence, Handong Global University, Pohang, Republic of Korea; Instituto Tecnologico Autonomo de Mexico, MEXICO

## Abstract

Successful new product development requires the integration of design and engineering, bridging the gap between technological feasibility and user-centric considerations. However, direct collaboration between designers and engineers with heterogeneous knowledge presents challenges. In this context, the role of design engineers—professionals skilled in both design and engineering—becomes pivotal. This study categorizes inventors into three primary groups: engineers, designers, and design engineers based on the type of patent applications they hold and investigates their differences in knowledge portfolios and collaboration patterns. The study relies on patent data for 4,665 US publicly-traded firms from 1980 to 2015 from the PATSTAT database, and constructs two networks for each firm period: a social network of inventors and a knowledge network of knowledge elements. Findings show that design engineers are highly connected within the social network but have disconnected knowledge in the knowledge network in comparison to engineers. While design engineers may not be the primary drivers of firms’ technological innovations, they facilitate interdisciplinary communication and decision-making, fostering a design-technology integrated new product development environment. This research has practical implications for firms seeking to optimize their innovation processes by creating interdisciplinary teams that harness the complementary strengths of engineers and design engineers.

## 1. Introduction

Successful new product development (NPD) hinges on fostering integrated product development through knowledge exchange and shared decision-making between designers and engineers [1, 2]. This holistic approach to NPD incorporates technological and user-centric considerations, ensuring that the resulting products are technically feasible and meet the target market’s needs [3]. However, direct knowledge sharing and collaboration between designers and engineers with heterogeneous knowledge frequently involves challenges and coordination costs [4]. Accordingly, design engineers—inventors who participate in both design and technology development—can play a pivotal role in bridging the gap between design and technology in the NPD process by facilitating mutual understanding and shared decision-making between designers and engineers. James Dyson, the founder of Dyson, remarked, “*At Dyson*, *there is no division between the engineers and the designers*. *We don’t have industrial designers*. *All our engineers are designers and all our designers are engineers* [5].” Engineers are increasingly confronted with the need to possess a broader skill set that encompasses both technical expertise and an understanding of design principles and user-centered thinking [2].

Design engineers specialize in design processes across various engineering and design disciplines. They typically collaborate with designers and engineers to develop conceptual and detailed designs that ensure product functionality and performance. Moreover, design engineers work closely with marketing teams to align product concepts with customer requirements and may supervise the NPD process. Investigating the role of design engineers is an intriguing topic, given the increasingly interdisciplinary nature of modern product development. However, in traditional firm innovation research, inventors are defined as a simple collection of engineers [6–9] or categorized into two groups—engineers and designers—to analyze their distinct roles and contributions to NPD [10–13].

This study takes a progressive step beyond prior research by categorizing inventors participating in NPD within a firm into three primary groups: engineers, designers, and design engineers. First, it provides new evidence on the differences in knowledge portfolios (i.e., combinatorial potential) between engineers and design engineers. Taking this step forward, this study examines and compares collaboration patterns among designers, engineers, and design engineers. Specifically, we investigate the role of design engineers as key collaborators and knowledge intermediaries, facilitating cross-functional decision-making within firms. While a few studies have explored aspects of their role, such as Hong et al. [14] on project oversight in the NPD process and Cross [15] on resolving tensions between designers and engineers, our study is the first quantitative empirical research that relies on patent data to identify design engineers within firms.

To this end, we retrieved patent data published by publicly traded firms headquartered in the United States between 1980 and 2015 from the PATSTAT database. We determined the job position of each inventor based on the type of patent application they held (i.e., design, utility patents, or both). The distinction between utility and design patents is rooted in the legal criteria for patent acquisition. A utility patent is granted to individuals who “*invent or discover any new and useful process*, *machine*, *manufacture*, *or composition of matter*”, while a design patent is granted to those who “*invent any new*, *original*, *and ornamental design for an article of manufacture*” [16]. Subsequently, we constructed two types of networks for each firm period: a social network comprising inventors and a knowledge network comprising knowledge elements [17–19]. We theorized and empirically evaluated how engineers and design engineers differ in degree and betweenness centralities within a firm’s knowledge and collaboration networks. The final sample comprises 1,576,880 inventor-period observations across 4,665 firms over 12 periods from 1980 to 2015.

The inventor-time fixed-effects regressions reveal that design engineers, compared to engineers, exhibit denser connections with other inventors in the social network but relative disconnection in the knowledge network. This nuanced role variation played by design engineers across different networks enhances our understanding of their dynamic organizational involvement. While design engineers may not be the primary drivers of technological innovation, they are essential as mediators and facilitators of knowledge transfer by leveraging their interdisciplinary position to disseminate knowledge and foster a design-engineering integrated NPD environment within the firm. These findings have significant implications for firms seeking to maximize the synergy between design and engineering in their innovation process, highlighting the valuable role that design engineers can play in facilitating cross-functional collaboration and decision-making in the NPD process and bridging the gap between market conceptualization and production practicalities.

This paper is organized as follows. Section 2 describes the theoretical background for the hypotheses regarding the distinct roles and contributions of designers, engineers, and design engineers in the NPD process. Section 3 presents the research design, explaining how the inventors’ knowledge and collaboration networks are constructed, other variables, and the method used for model specification. Section 4 presents the empirical test findings. Finally, Section 5 summarizes the research and its contributions and outlines plans for future research.

## 2. Literature review and hypotheses

### 2.1 Inventors’ roles in new product development

Owing to the exponential growth of scientific and technological knowledge, the expertise required for NPD has become highly specialized among multiple inventors [20, 21]. In modern technology and science, fields have become increasingly complex, spanning various domains. The development of new products requires in-depth knowledge and skills in diverse areas. It is challenging for a single individual to acquire expertise across all these domains, leading experts to focus on highly specialized fields [22]. In this context, NPD often requires effective communication and collaboration among teams of experts with field-specific knowledge and skills [2]. Each expert contributes specialized knowledge to their respective fields, enabling firms to combine their expertise to drive innovation in product development.

In traditional firm innovation research, inventors or product developers are either defined as engineers or broadly categorized into two groups—engineers and designers—to analyze their distinct roles and contributions to NPD. Kobayashi and Watanabe [12] conducted an empirical investigation into the capability of industrial designers to drive technological innovations. Similarly, Hong et al. [14] examined the role of design engineers in NPD, revealing their contributions to setting clear project targets and enhancing product development productivity. The drivers of a firm’s product innovation cannot be exclusively confined to engineers. This study takes a progressive step beyond prior research by categorizing inventors participating in NPD within a firm into three groups: engineers, designers, and design engineers. Each inventor group possesses a distinct knowledge base and contributes uniquely to NPD.

Engineers use their specialized technical knowledge to develop a product’s operation and functionality, considering both technical constraints and functional requirements (e.g., durability, stability, and performance). Designers primarily operate in visual, graphic, and industrial design fields. They are mainly responsible for visual design elements such as products, websites, and logos, and may also be involved in user interface and user experience design. Designers primarily focus on designing the aesthetic appearance of a product, including its form, color, and patterns [23]. Design Engineers participate in both design and technical development, bridging the development and market sides of a product during the NPD process. From a developmental perspective, they collaborate with engineering teams to develop designs that consider technical constraints, or solve engineering problems using design thinking. From a market perspective, they work with various teams, like design and marketing teams, to enhance the user experience and product design elements. Based on their understanding of the functioning principles of the product, they may also change the product design by considering the functionality-usability balance [3].

### 2.2 Knowledge portfolio of engineers and design engineers

An inventor’s knowledge base is shaped and developed through on-the-job learning experiences and engagement in inventive activities [17]. Design engineers may come from diverse educational backgrounds, such as industrial design and engineering. However, because they participate in both design and technology development activities in the workplace, they tend to cultivate specialized knowledge distinct from traditional engineers [2]. Their expertise covers design (market side) and technical knowhow (development side). From the perspective of knowledge specialization, engineers typically possess a comprehensive central knowledge base within firms because of their focused training. However, design engineers often lack the same level of technical expertise found in engineers and focus on specific knowledge domains where the convergence of design and technology is essential.

Inventors’ search for innovation involves combining and recombining various knowledge elements within their knowledge bases [24, 25]. The effectiveness of their search activities depends on the combinatorial potential of their knowledge elements within the inventor’s specific knowledge domain and extended combinatorial opportunities across other knowledge domains, expanding into different areas of knowledge [17]. In this context, a knowledge domain refers to the overarching field or subject area within which an inventor operates, while a knowledge element pertains to specific components within that domain, which inventors combine or recombine during their search for innovation. The inventor’s combinatorial capability is linked to the structural characteristics of their knowledge elements within the firm’s knowledge network [26, 27]. For instance, the degree centrality of a knowledge element within a firm’s knowledge network, which indicates the number of knowledge elements connected to the focal knowledge element, represents its potential for combinations with other knowledge elements. A pair of knowledge elements combined in previous inventions is more likely to successfully recombine than a random pair of unrelated knowledge elements. Therefore, researchers with knowledge elements of high degree centralities tend to have greater combinatorial potential. Additionally, the betweenness centrality of a knowledge element indicates its potential for combinations with knowledge elements in other knowledge domains. A knowledge element with high betweenness centrality has the potential to serve as a brokering knowledge between knowledge elements in different knowledge domains that are themselves disconnected. A researcher with the brokering knowledge element is more likely to have greater combinatorial opportunities in knowledge domains that have not yet been explored but are open for future investigations. From this perspective, the structural position of an inventor’s knowledge elements determines the opportunities and constraints for combinations, thereby influencing the outcomes of their search for innovation [26, 27].

We compared the knowledge base structures of engineers and design engineers within a firm’s knowledge network. They are likely to exhibit differences in the composition of their knowledge elements, which vary in terms of their combinatorial potential [17, 28]. These differences stem from the distinct roles engineers and design engineers play in NPD. Engineering roles often involve tackling complex technical problems and seeking technical innovation, which necessitates the combination and recombination of diverse knowledge elements. Consequently, engineers are likely to possess a higher combinatorial potential than design engineers, resulting in higher degree and betweenness centralities of their knowledge elements within the firm’s knowledge network. Conversely, the role of design engineers is to comprehensively integrate design (product marketability) and technology (technical feasibility) in the NPD process to enhance product-market fit. Therefore, design engineers may have limited technical knowledge and lower recombination capabilities compared to engineers. Thus, we propose the following hypotheses:

***Hypothesis 1A*.** *The average degree centrality of a researcher’s knowledge elements in a firm’s knowledge network is higher for engineers than for design engineers*.
***Hypothesis 1B*.** *The average betweenness centrality of a researcher’s knowledge elements in a firm’s knowledge network is higher for engineers than for design engineers*.

### 2.3 Collaboration between designers and engineers

Integrating product development through knowledge exchange and shared decision-making between designers and engineers is essential for successful product development [13, 29]. Collaboration between designers and engineers promotes a holistic approach to NPD that incorporates technological and user-centric considerations [3]. In this process, designers evaluate the feasibility of their design concepts from a technical perspective, whereas engineers elucidate technical constraints and provide insights into how functional requirements can be achieved. Numerous studies have provided empirical evidence supporting the integration of designers and engineers into the NPD process. Gemser and Leenders [30] found that incorporating industrial design into the NPD process is associated with improved firm performance. More recently, Kobayashi and Watanabe [12] discovered that involving designers in technology development activities enhances inventive performance. Chen et al. [29] contended that effective management and sharing of engineering knowledge throughout the NPD process are essential for successful product design. Similarly, Bogers and Horst [3] emphasized the significance of engaging a broader range of NPD stakeholders in early-stage prototyping, which is critical in determining a product’s functional specifications.

Traditionally, ‘form follows function’ suggests that design is typically incorporated into the NPD process after the development of technology [31]. However, this approach, in which technology comes first, followed by design, tends to emphasize the technical aspects of a product and may restrict the development of user-oriented products [11, 32]. Considering the design in the later stages of NPD can lead to significant costs and time for product modifications and further improvements [14, 33, 34]. The successful integration of design and technology entails adopting a comprehensive approach to product development, considering technical feasibility and marketability from the outset [30]. This ensures that the resulting products are not only technically feasible but also meet market demands and customer preferences. To achieve this, effective communication and collaboration between designers and engineers are essential.

Direct knowledge sharing and collaboration between designers and engineers often involves numerous challenges and coordination costs. This aligns with previous research highlighting the difficulties in knowledge sharing among individuals with heterogeneous knowledge [4, 22]. Previous studies have delineated how designers and engineers differ in their behaviors and capabilities. For instance, designers and engineers may take different approaches to problem-solving because of their expertise in distinct fields. This may have led to conflicting results. Designers often prioritize aesthetics, ergonomics, usability, safety, and user experience, focusing on the interactions between users and products during development. They are primarily concerned with how design affects users and their perceived value. In contrast, engineers concentrate on the functional aspects of design, such as functionality and performance requirements, and the design of the internal components necessary for a product’s operation [23].

Designers approach problems creatively and intuitively, whereas engineers emphasize analysis and logic and seek technical solutions. According to Bonsiepe [35], designers primarily consider “*designability*” and aim to provide “*new experiences for people*,” whereas engineers concentrate on creating “*new knowledge*.” Similarly, Krippendorff [36] suggests that designers are interested in “*what will exist and is unobservable*,” while engineers focus on “*what already exists and is observable*.” These differences are also evident in their ability to generate creative ideas. Agogué et al. [10] compared the creative behaviors of designers and engineers and found that designers tend to be less fixated and create more ideas than engineers. Yu et al. [13] analyzed their differences in prototyping behavior, revealing that engineers concentrate on testing whether product features and functions meet user requirements in actual user contexts, whereas designers explore new design possibilities using various materials and tools.

Designers and engineers have distinct collaborative cultures. Chan et al. [37] pointed out that the non-decomposable nature of design, in which the entire product is designed holistically rather than as individual components, leads to significant coordination costs when working in teams. Conversely, technology is relatively decomposable. Furthermore, it is challenging for an engineer to possess all the technical knowledge required to develop an idea into a product. In such cases, the benefits of knowledge diversity outweigh the coordination costs of collaboration. Therefore, most technological innovations originate from teamwork rather than from a single inventor [38]. Regarding diversity in collaboration, engineers often assume more specialized roles and frequently collaborate with inventors within their specific fields. By contrast, designers must engage with numerous teams involved in the NPD process, including mechanical, software, and hardware engineering teams, to design various aspects of a product.

### 2.4 Design engineers in a firm’s collaboration network

Design engineers, equipped with both design capabilities (i.e., design thinking and creativity) and technical knowledge, play a crucial role in facilitating communication and collaboration between designers and engineers. Typically, they engage in various stages of the NPD process, ranging from concept development to prototype creation, thereby contributing significantly to the overall success of NPD [14]. Design engineers take on the overall coordination of NPD to promote product development that integrates visual, technical, and functional aspects [39]. For instance, during the concept development and planning phases, design engineers combine design creativity with engineering knowledge to generate new ideas and explore ways to realize them.

Throughout the design implementation process, design engineers share technical constraints and manufacturing requirements with designers to enhance product feasibility and marketability (e.g., cost). Notably, Hong et al. underscored the role of design engineers in overseeing and coordinating projects across the entire NPD process. They support product development teams by clarifying objectives and converging and sharing knowledge among team members. In cross-functional NPD processes, design engineers contribute to improving productivity during product development. Hence, design engineers are likely to be positioned within firms as key collaborators or knowledge providers to coordinate the critical elements of NPD and participate in cross-functional decision-making. Simply put, design engineers collaborate with various inventors participating in NPD to promote knowledge sharing within firms. Furthermore, they are likely to have significant influence and responsibilities within firms.

By leveraging their dual expertise in design and technical aspects, design engineers can consider the role of mediators between designers and engineers. Cross [15] proposed that design engineers significantly mitigate frequent conflicts between designers and engineers, thus promoting cross-functional decision-making. Moreover, Hong et al. [14] noted that design engineers, given their unique position within NPD, effectively bridge the gap between market conceptualization and production practicalities.

A firm can be conceptualized as having two interconnected networks: a knowledge network comprising knowledge elements and a social network comprising inventors [18]. The social network within a firm mirrors collaboration among inventors. We examine the significance and roles of inventors based on their structural positions within firms’ collaboration networks. The structure of this collaborative network is closely linked to an individual’s access to information, influence, and opportunities for new collaborations [26, 40, 41]. For instance, inventors with high degree centrality are directly connected to many other inventors, which enables them to access more knowledge and disseminate information more broadly [42, 43].

An inventor’s betweenness centrality is the proportion of all shortest paths between pairs of other inventors in the network that pass through that inventor [44]. Thus, a high betweenness centrality value signifies an inventor’s role as a mediator or broker among other inventors. This implies that they have some control over interactions between other inventors and the dissemination of knowledge within the network [45, 46]. Previous research on collaborative networks employed betweenness centrality to measure an individual’s intermediary role. Yan and Ding [47] proposed that authors with high betweenness centrality in a co-authorship network link groups from different research fields, suggesting their engagement in interdisciplinary research.

Both degree and betweenness centralities measure a node’s prominence and influence within a network; however, they rely on different measurement methods and underlying assumptions. Degree centrality gauges a node’s centrality by considering only the node’s local structure, not the global network structure. Conversely, betweenness centrality factors in the network structure and the relationships between nodes, encompassing information about the overall network configuration. A node with high degree centrality does not necessarily hold a strategically significant position throughout the entire network.

In the literature, various centrality measures (such as degree, closeness, betweenness, and eigenvectors) have been utilized in combination to identify the importance of nodes based on their structural positions within a network [43, 48]. Das et al. [46] compared multiple centrality measures and proposed distinct applications for each. Degree centrality is effective for identifying nodes directly impacting other nodes, whereas betweenness centrality is used to identify nodes mediating information among multiple nodes. According to Dias et al. [49], degree centrality, which indicates how well a node is connected, may not provide insights into how strategically positioned that node is within the network. However, this illustrates the activity and ability of a node to build collaborative links. Yet, betweenness centrality reveals how much a node functions as a gatekeeper, connecting other nodes, and signifying its capacity to control information and other resources.

Beaudry and Schiffauerova [43] employed the degree and betweenness centralities of inventors within a firm’s co-patent network to identify central inventors and those serving intermediary roles within the firm. Consequently, these two centrality measures significantly affected the quality of inventors’ invention outputs. In line with Beaudry and Schiffauerova [43], we compare inventors’ degree and betweenness centralities within a firm’s collaborative network to assess the strategic positions of engineers and design engineers. Specifically, we anticipate that design engineers play a pivotal role in the NPD process by collaborating with a broad range of product development teams within a firm. This collaboration facilitates interdisciplinary knowledge sharing and project coordination. Thus, we propose the following hypotheses:

***Hypothesis 2A*.** *The degree centrality of a researcher in a firm’s collaboration network is higher for design engineers than for engineers*.
***Hypothesis 2B*.** *The betweenness centrality of a researcher in a firm’s collaboration network is higher for design engineers than for engineers*.

## 3. Data and methods

### 3.1 Data and sources

To test our hypotheses, we utilized utility patent data from the DISCERN database as our reference dataset [50]. The DISCERN database is restricted to all US-headquartered publicly traded firms that hold at least one patent and report positive research and development (R&D) expenses for at least one year between 1980 and 2015. We then extracted the design patents of the sample firms from the PATSTAT database. Our analysis incorporated 1,382,439 patent applications (1,260,045 utility patents and 122,394 design patents) filed between 1980 and 2015, associated with 4,665 firms. Design and utility patents are two distinct types of patent applications, each serving different purposes in safeguarding inventions. Design patents protect the appearance or ornamentation of a product, while utility patents protect the functional aspects of an invention [16]. Our empirical study focused on inventors as the primary unit of analysis. To provide a more comprehensive view of inventors’ collaboration and knowledge dynamics, we divided this dataset into 12 distinct periods, each covering three years.

For our analysis, we first obtained inventor information on design and utility patents from the PATSTAT database. Second, we classified the inventors into three categories based on the types of patent applications they held: designers, engineers, and design engineers. We considered an inventor to be a designer if they held only design patents, an engineer if they held only utility patents, and a design engineer if they held both design and utility patents. Once classified as design engineers, their categorization remained consistent throughout the sample period as long as they continued to engage in patenting activities within the same firm.

During this process, we encountered a situation where design engineers were initially labeled as either designers or engineers, owing to two distinct person identification numbers for design and utility patents. To address this issue, we standardized their personal IDs by categorizing inventors as design engineers only if they shared the same name and obtained design and utility patents from the same firm. Another challenge arose when some inventors had the same ID, but held patents associated with multiple firms. Approximately 4.9% of inventors fall into this category, which could be attributed to factors such as changes in firm name, organizational restructuring (e.g., mergers and acquisitions), joint R&D, and inventor mobility. We excluded these inventors from the sample for two reasons. First, a patent with multiple assignees may indicate inter-firm collaboration (i.e., joint R&D), which is beyond the scope of this study. Second, determining an inventor’s precise affiliation is complex as it may involve various forms of collaboration (i.e., joint R&D, outsourcing, and licensing) or inventor mobility. Consequently, we obtained a final dataset comprising 73,808 designers, 960,961 engineers, and 15,357 design engineers.

**Fig 1:**
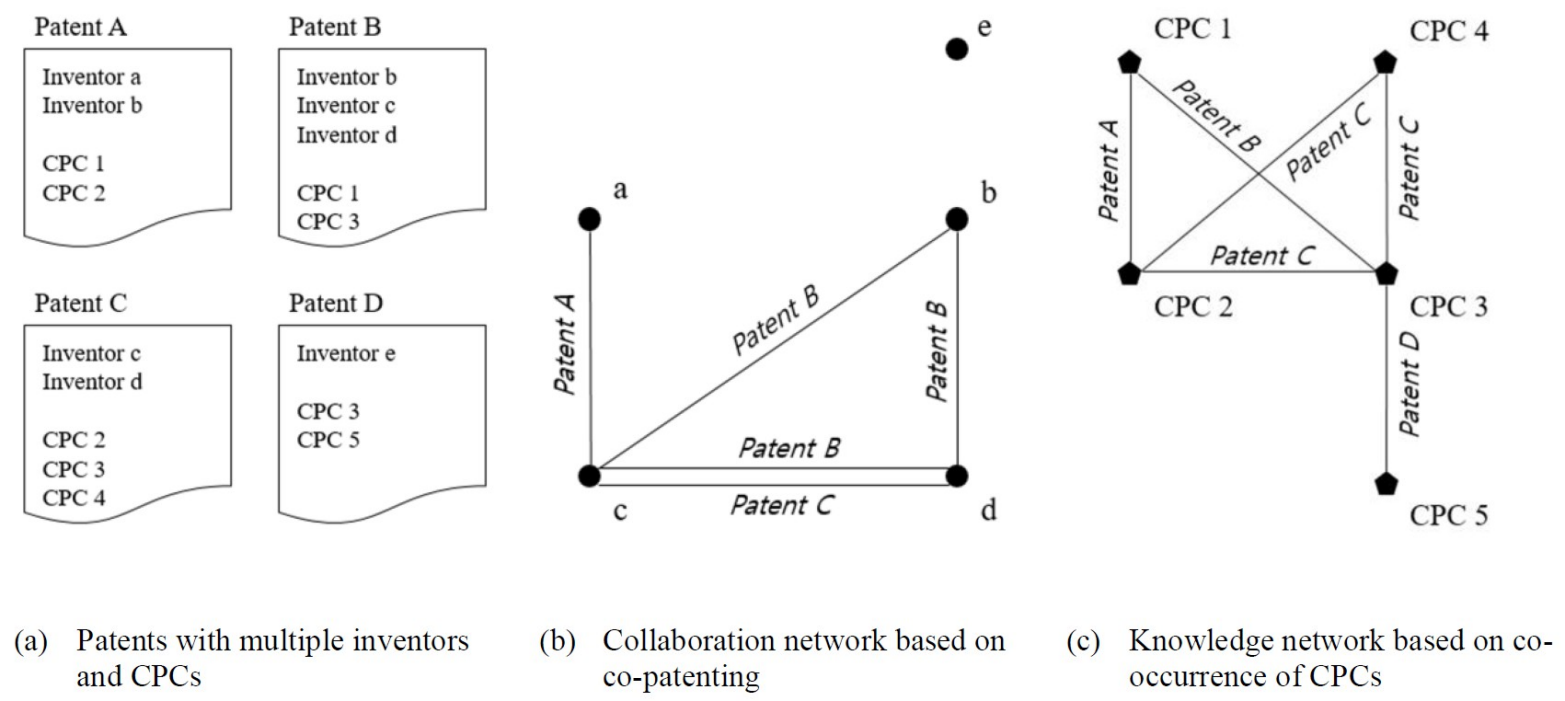
Illustration of constructing collaboration and knowledge networks.

We constructed a collaborative network based on co-patenting among inventors [26, 41, 51]. In this approach, each inventor represents a node and ties indicate collaboration on a patent application within the same firm (see Fig 1(A) and 1(B) for an illustration of our collaboration network construction). These ties were weighted to account for the depth and intensity of collaboration, measured by the number of co-patents between inventors. We generated networks for each firm period to capture collaboration dynamics over time. Our primary focus is to assess the centrality of each inventor within these networks. This centrality analysis provides insights into inventors’ relative importance and influence on their collaborative invention activities, revealing the firm’s key collaborators and knowledge mediators.

In addition to the collaboration networks, we developed knowledge networks for each firm period based on Cooperative Patent Classification (CPC) codes (see Fig 1(A) and 1(C) for a visual representation of our knowledge network construction) [19, 52]. An inventor’s knowledge elements were defined as all 4-digit CPC codes related to their patents, with each CPC code represented as a node and a tie indicating the co-occurrence of CPC codes within a patent application [19]. It is important to note that designers are excluded from the knowledge network analysis as design patents do not have CPC codes. Therefore, we specifically focus on the comparison between engineers and design engineers who possess technological knowledge elements represented by CPC codes. In the knowledge network analysis, we focused on the centrality of each inventor’s knowledge elements within the firm’s knowledge network. After creating collaboration and knowledge networks for each firm period, we computed the centrality measures at the inventor level, including degree and betweenness centralities. For knowledge networks, because inventors may have multiple CPC codes, we calculated the average centrality of each inventor’s knowledge elements to measure their knowledge centrality. The final sample comprises 1,022,219 inventors and 1,576,880 inventor-period observations from 1980 to 2015.

### 3.2 Variables

#### Dependent variables

*Knowledge network degree centrality* (KNOW_DEG_it_). The degree centrality of individual inventors within a firm’s knowledge network is determined by calculating the average degree centrality of the knowledge elements associated with each inventor. This process involved two steps. Initially, we assessed the degree centrality, which refers to the sum of the weights of all interconnected knowledge elements for each knowledge element present in the firm’s knowledge network during specific time periods. Subsequently, we sorted the collection of knowledge elements attributed to each inventor and calculated the average degree centrality across these knowledge elements. Eq (1) shows the equation for the knowledge network degree centrality of inventor *i* in a knowledge network.

$$Knowledge\;Degree\;Centrality(i)=\frac{1}{K(i)}\sum_{k\in K(i)}\left(\sum_{q\in G_{K}}w_{kq}\right)$$ (1)

where, in a knowledge network denoted as *G*_*K*_, K(*i*) refers to the number of all knowledge elements associated with inventor *i* and *k* represents a knowledge element in the set K(*i*). *w*_*kq*_ is the weight of the link between knowledge element *k* and its neighboring knowledge elements, denoted by *q*.

*Knowledge network betweenness centrality* (KNOW_BTW_it_). The computation of betweenness centrality for each inventor within a firm’s knowledge network follows a similar approach. We calculated the average betweenness centrality across all the knowledge elements associated with each inventor. This reveals the extent to which inventors’ knowledge elements act as intermediaries between pairs of knowledge elements.


$$Knowledge\;Betweenness\;Centrality(i)=\frac{1}{K(i)}\sum_{k\in K(i)}\left(\sum_{s\neq k\neq t}\frac{\sigma_{st}(k)}{\sigma_{st}}\right)$$ (2)

In Eq (2), *s* and *t* are distinct pairs of knowledge elements in the network and *σ*_*st*_ is the total number of shortest paths between knowledge elements *s* and *t*. *σ*_*st*_(*k*) is the number of those shortest paths that pass through knowledge elements *k*.

*Collaboration network degree centrality* (COLLAB_DEG_it_). Degree centrality was calculated for the collaboration network described in the previous subsection to test Hypothesis 2A. To calculate the degree centrality for a weighted collaboration network, *G*_*C*_, where *w*_*ij*_ represents the link between inventors *i* and *j*, the weight *w*_*ij*_ is zero if inventors *i* and *j* are disconnected. The degree of inventor *i* is determined by summing the weights of all links between inventor *i* and its neighboring inventors, denoted by *j* [53]. Simply put, the degree of a node quantifies the total strength of the connections that inventor *i* has with their neighbors. The degree centrality of inventor *i* in a collaborative network is defined as:

$$Collaboration\;Degree\;Centrality(i)=\sum_{j\in G_{C}}w_{ij}$$ (3)

*Collaboration network betweenness centrality* (COLLAB_BTW_it_). The betweenness centrality of each inventor in a firm’s collaboration network is used to test Hypothesis 2 B. The betweenness centrality of inventor *i* is calculated by determining how often *i* acts as an intermediary along the shortest paths between the other pairs of inventors within the network. This is achieved by quantifying the fraction of the shortest paths that pass through *i* out of all possible shortest paths between other inventor pairs. In simpler terms, an inventor’s betweenness centrality captures the extent to which the inventor serves as a bridge for communication between other inventors. The betweenness centrality of inventor *i* in the collaborative network was calculated as follows:

$$Collaboration\;Betweenness\;Centrality(i)=\sum_{m\neq i\neq n}\frac{\sigma_{mn}(i)}{\sigma_{mn}}$$ (4)

where *m* and *n* are distinct pairs of inventors in the network and *σ*_*mn*_ is the total number of shortest paths between inventors *m* and *n*. *σ*_*mn*_(*i*) is the number of those shortest paths that pass through inventor *i*. This equation quantifies how often inventor *i* acts as a bridge along the shortest paths between inventor pairs within the collaboration network. The higher the betweenness centrality value, the more influential *i* is in connecting other inventors in the network.

#### Independent variable (POSITION_it_)

We included a dummy variable indicating the inventor’s job position. As explained in the previous subsection, we determined individual inventors’ job positions based on the types of patent applications they possessed up to period t. In this context, we created a dummy variable, POSITION_*it*_, which takes a value of 0, 1, or 2 depending on whether a person holds only utility patents, both technology and design patents, or only design patents. This distinction is made for engineers, design engineers, and designers.

#### Control variables

*Individual-level control variables*. The inventors’ structural positioning within a firm’s collaboration and knowledge networks can be influenced by their invention capabilities. To account for these influences, we incorporated several individual-level control variables. First, we controlled for an inventor’s invention productivity (PRDT), defined as the total number of design and utility patents [54]. Second, we considered invention quality (QLT), which indicates the impact and significance of an inventor’s patents [55, 56]. This measure assesses the number of citations received for an inventor’s technology and design patents within five years after publication. Third, we included invention reputation (RPT), defined as the number of forward citations of an inventor’s patents cited explicitly by other inventors within the same firm. This captures inventors’ recognition and influence among their colleagues [17]. Finally, we incorporated inventor tenure (TENURE), representing the time from an inventor’s initial appearance in the patent dataset to the given period [54]. This control variable accounts for an inventor’s experience in the field of invention.

*Firm-level control variables*. In addition to individual-level factors, we incorporated two firm-level control variables that could affect the size and characteristics of collaboration and knowledge networks. For instance, in a firm with a larger number of patents, individual inventors are more likely to specialize in certain knowledge domains than inventors in a firm with fewer patents. Accordingly, we included an indicator of a firm’s inventive output, firm productivity (FIRM_PRDT), which is determined by the total number of design and utility patents generated by the firm. Furthermore, we included the number of employees (EMP), which serves as a proxy for firm size, to control for the firm’s capacity to undertake extensive R&D activities [57]. The variables used in the analyses are presented in Table 1.

**Table 1:** Description of variables.

	Variables	Abbreviation	Description
Dependent variables	Knowledge network degree centrality	*KNOW*_*DEG*_*it*_	Average degree centrality of an inventor’s knowledge elements in a firm’s knowledge network
	Knowledge degree betweenness centrality	*KNOW*_*BTW*_*it*_	Average betweenness centrality of an inventor’s knowledge elements in a firm’s knowledge network
	Collaboration network degree centrality	*COLLAB*_*DEG*_*it*_	Degree centrality of an inventor in a firm’s collaboration network
	Collaboration network betweenness centrality	*COLLAB*_*BTW*_*it*_	Betweenness centrality of an inventor in a firm’s collaboration network
Independent variable	Dummy for engineers, design engineers, and designers	*POSITION*_*it*_	A dummy variable that takes the value of 0, 1, 2 if a person has patented only utility patents, both technology and design patents or only design patents, respectively
Control variables	Invention productivity	*PRDT*_*it*_	An inventor’s total number of design and utility patents
	Invention quality	*QLT*_*it*_	Number of citations on an inventor’s design and utility patents
	Invention reputation	*RPT*_*it*_	Number of citations on an inventor’s design and utility patents by other inventors in the firm
	Inventor tenure	*TENURE*_*it*_	Years to time *t* since the inventor first appeared in the patent dataset
	Firm productivity	*FIRM*_*PRDT*_*it*_	A firm’s total number of design and utility patents
	Firm size	*FIRM*_*SIZE*_*it*_	Number of employees in a firm

### 3.3 Empirical model

From the analysis of intrafirm collaboration and knowledge networks, we constructed a comprehensive panel dataset consisting of 1,576,880 inventor-period observations across 4,665 firms over the period from 1980 to 2015. To isolate the distinct disparities between engineers and design engineers, while simultaneously controlling for latent variations across inventors and periods, we employed a two-way fixed-effects approach. This method was designed to account for specific fluctuations inherent to each inventor and time period. Employing inventor-time fixed effects can mitigate potential biases stemming from omitted variables and unobservable heterogeneity. We also conducted a Hausman test to assess the appropriateness of using fixed effects rather than random effects. The results of the Hausman test indicated that the fixed-effects approach was more suitable. Accordingly, a panel fixed-effects regression model is constructed as follows:

$$Y_{it}=\alpha +\beta_{n}{Dummy}_{int}+\beta_{n}{Control}_{int}+\mu_{i}+{\;\lambda }_{t}+\epsilon_{it}$$ (5)

where subscript *i* refers to an inventor and *t* refers to the time periods. *μ*_*i*_ and *λ*_*t*_ represent the unobserved individual and time effects, respectively. Eq (5) is estimated for the four dependent variables: knowledge degree centrality, knowledge betweenness centrality, collaboration degree centrality, and collaboration betweenness centrality. Additionally, all variables included in our estimation analysis were transformed into logarithmic forms to account for the right-skewed distributions of the variables.

## 4. Results

### 4.1 Descriptive statistics

Table 2 presents the variables’ descriptive statistics and their correlation matrices. There is a weak correlation between the two centrality measures—degree and betweenness centralities—for both collaboration and knowledge networks. Interestingly, the knowledge degree centrality and collaboration betweenness centrality are highly correlated. This suggests a relationship between inventors’ role as a bridge in collaborative networks and the importance of their knowledge in the firm.

**Table 2:** Descriptive statistics and correlations of variables.

Variables		1	2	3	4	5	6	7	8	9	10
1	KNOW_DEG	1.00									
2	KNOW_BTW	0.17	1.00								
3	COLLAB_DEG	0.15	0.11	1.00							
4	COLLAB_BTW	0.72	0.19	0.28	1.00						
5	PRDT	-0.01	0.04	0.13	0.02	1.00					
6	QLT	0.01	0.02	0.07	0.02	0.36	1.00				
7	RPT	-0.00	0.02	0.06	0.01	0.26	0.29	1.00			
8	TENURE	-0.02	0.00	0.05	-0.02	0.16	-0.04	0.03	1.00		
9	FIRM_PRDT	-0.07	-0.04	-0.02	-0.09	0.07	0.01	-0.03	0.08	1.00	
10	FIRM_SIZE	-0.15	-0.08	-0.06	-0.19	0.01	-0.01	-0.00	0.03	0.24	1.00
Min		0	0	0	0	1	0	0	0	1	0
Max		3	0.5	1	3	517	68191	6814	45	5,854,301	841.1
Mean		0.02	0.00	0.00	0.04	2.15	18.14	3.87	3.18	193,360.2	100.35
S.D.		0.10	0.00	0.02	0.13	3.83	87.04	22.19	4.43	563,366.9	125.82

Multicollinearity was assessed using a variation inflation factor (VIF) test conducted on all the variables. The VIF ranges from 1.57 to 1.71, averaging at 1.64. All VIF values were below the commonly accepted threshold of four, indicating that significant multicollinearity concerns did not arise from the analysis [58].

### 4.2 Regression analysis

Table 3 reports the two-way fixed-effects regression results for knowledge and collaboration network centralities. Models 1 to 4 present the outcomes for knowledge network centralities, while Models 5 to 8 display the findings for collaboration network centralities. In Models 2 and 4, the coefficients associated with the job position dummy for design engineers exhibit the expected negative signs, indicating that design engineers are associated with knowledge elements characterized by lower degree and betweenness centralities within a firm’s knowledge network than engineers. These findings support Hypotheses 1A and 1B. This alignment with our expectations sheds light on distinct patterns regarding the positioning of design engineers within a firm’s knowledge network. Design engineers tend to exhibit lower combinatorial potential within a firm’s knowledge network, as evidenced by their lower degree centrality compared to engineers. Additionally, the lower betweenness centrality of the design engineers suggests that they are positioned less frequently as intermediaries or bridges to facilitate the recombination of knowledge between different knowledge domains. This implies that their role may be more localized within specific domains of expertise rather than encompassing broader connections with various knowledge segments. By contrast, engineers are characterized by greater combinatorial opportunities not only within the focal domain of knowledge but also in domains that remain unexplored.

**Table 3:** Two-way fixed-effects regression models for collaboration and knowledge network centrality measures.

		Knowledge network				Collaboration network			
		(1)	(2)	(3)	(4)	(5)	(6)	(7)	(8)
		DEG	DEG	BTW	BTW	DEG	DEG	BTW	BTW
Control variables	PRDT	0.337***	0.342***	0.077***	0.077***	0.474***	0.476***	0.397***	0.396***
		(0.001)	(0.001)	(0.001)	(0.006)	(0.001)	(0.001)	(0.001)	(0.001)
	QLT	0.003***	0.003***	-0.000*	-0.000**	0.002***	0.001***	0.006***	0.006***
		(0.000)	(0.000)	(0.000)	(0.000)	(0.000)	(0.000)	(0.000)	(0.000)
	RPT	-0.004***	-0.004***	0.000***	0.000***	0.001***	0.001***	0.006***	0.006***
		(0.000)	(0.000)	(0.000)	(0.000)	(0.000)	(0.000)	(0.000)	(0.000)
	TENURE	-0.003***	-0.004***	-0.000	-0.000	-0.003***	-0.003***	-0.005***	-0.005***
		(0.000)	(0.000)	(0.000)	(0.000)	(0.000)	(0.000)	(0.000)	(0.000)
	FIRM_PRDT	-0.178***	-0.177***	-0.012***	-0.012***	-0.001	-0.001	-0.188***	-0.188***
		(0.001)	(0.001)	(0.000)	(0.000)	(0.001)	(0.001)	(0.001)	(0.001)
	FIRM_SIZE	-0.255***	-0.253***	-0.032***	-0.032***	-0.023***	-0.025***	-0.275***	-0.275***
		(0.002)	(0.002)	(0.001)	(0.001)	(0.003)	(0.003)	(0.002)	(0.002)
Dummy variables	DESIGN_ENGINEER		-0.582***		-0.049***		0.150***		0.122***
			(0.009)		(0.004)		(0.012)		(0.008)
	DESIGNER						-0.034**		0.133***
							(0.015)		(0.010)
	Constant	-2.820***	-2.849***	-6.558***	-6.560***	-7.024***	-7.015***	-2.456***	-2.457***
		(0.010)	(0.010)	(0.005)	(0.005)	(0.013)	(0.013)	(0.009)	(0.009)
	Observations	1,498,586	1,498,586	1,498,586	1,498,586	1,576,880	1,576,880	1,576,880	1,576,880
	Group	966,199	966,199	966,199	966,199	1,022,219	1,022,219	1,022,219	1,022,219
	VIF	1.71	1.61	1.71	1.61	1.66	1.57	1.66	1.57
	Hausman test	8769.86***	15474.62***	283.53***	315.28***	2425.89***	1292.47***	10585.15***	10697.67***

Notes

* p < 0.1

** p < 0.05

*** p < 0.01. Standard errors are given in parentheses below the coefficients.

In Models 6 and 8, the coefficients of the design engineer dummy variable are statistically positive. This signifies that design engineers tend to possess higher degree and betweenness centralities than engineers in a firm’s collaboration network. These results empirically support Hypotheses 2A and 2B. Drawing insights from these outcomes, we can infer that design engineers are inclined to play integral roles in facilitating connections and serve as intermediaries within a firm’s collaboration network. This aligns with the expectations set forth by the hypotheses, indicating that design engineers’ unique skillsets contribute to their heightened centrality within the collaboration network.

Interestingly, the coefficient of the designer dummy is statistically negative in Model 6, implying that designers have a lower degree centrality than engineers in the collaboration network. This can be attributed to the non-decomposable nature of design [37]. When designing products, the focus is on the holistic design of the entire product, rather than on individual components. This holistic approach requires significant coordination when working in teams, leading to a lower degree centrality than engineers. By contrast, in Model 8, the coefficient of the designer dummy shows a positive sign, indicating that designers have a higher betweenness centrality than engineers in the collaboration network. One possible explanation could be that designers tend to collaborate with various teams due to the distinct characteristics of their engagement in design activities, from the physical appearance to the user interface and overall user experience of products. While designers may engage in fewer collaborative interactions than engineers, higher betweenness centrality signifies their crucial role in multidisciplinary collaboration. However, engineers tend to collaborate with others primarily within their specialized engineering domains, often interacting with colleagues who share similar technical expertise.

### 4.3 Robustness checks

**Fig 2:**
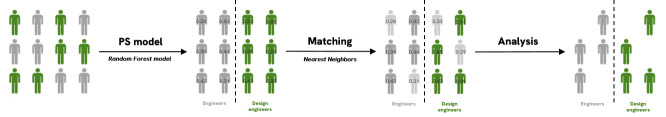
Illustration of the PSM process.

For robustness checks, we conducted a propensity score matching (PSM) to address potential confounding bias and further validate the findings from our main analysis. Given the substantial differences in the number of samples among engineers, designers, and design engineers, this technique is beneficial for ensuring that groups are balanced and comparable regarding key covariates. PSM involves estimating the probability of group assignment (propensity scores) for each individual, conditional on observed covariates, and subsequently matching these individuals based on their estimated scores [59]. To perform PSM across the three groups (engineers, designers, and design engineers), we first estimated the probabilities of individual inventors belonging to each group using a Random Forest (RF) model. The RF model is a powerful tool for assessing propensity scores because it captures the complex and nonlinear relationships between covariates and group assignments. In our RF model, we employed the control variables included in the previous estimation as covariates, which could have introduced bias into our analysis. These predicted probabilities then served as our propensity scores, indicating the likelihood of an individual being part of a specific group given their observed covariates. Building on this, we employed the Nearest Neighbors (NN) algorithm—the greedy 1:1 matching—to match individuals across the three groups using the estimated propensity scores. The NN algorithm identifies individuals across the three groups with the most similar propensity scores, thereby creating balanced comparison groups that share similar characteristics, enhancing the validity and reliability of our comparisons. Fig 2 illustrates the PSM process employed in this study. The final sample for robustness checks includes 10,293 inventors with an equivalent number of engineers, designers, and design engineers.

Table 4 presents the generalized least squares regression analysis results conducted on the PSM dataset. Notably, these findings are consistent with previous estimations as evidenced by the emergence of negative and statistically significant coefficients associated with the design engineer dummy in the models pertaining to knowledge network degree and betweenness centralities. Furthermore, positive and statistically significant coefficients are associated with the design engineer dummy in the models related to the collaboration network degree and betweenness centralities.

**Table 4:** Generalized least square regression analysis using PSM models.

		Knowledge network				Collaboration network			
		(1)	(2)	(3)	(4)	(5)	(6)	(7)	(8)
		DEG	DEG	BTW	BTW	DEG	DEG	BTW	BTW
Control variables	PRDT	0.251*** (0.017)	0.260*** (0.017)	0.055*** (0.004)	0.057*** (0.004)	0.299*** (0.008)	0.301*** (0.008)	0.359*** (0.009)	0.342*** (0.009)
	QLT	0.011*** (0.004)	0.011*** (0.004)	0.002** (0.001)	0.002** (0.001)	0.007*** (0.001)	0.004** (0.001)	-0.001 (0.001)	0.0005 (0.002)
	RPT	0.001 (0.003)	0.001 (0.003)	0.004*** (0.001)	0.004*** (0.001)	0.021*** (0.002)	0.019*** (0.002)	0.015*** (0.002)	0.016*** (0.002)
	TENURE	-0.003 (0.006)	-0.002 (0.006)	0.002 (0.002)	0.002 (0.002)	0.006*** (0.003)	0.003 (0.003)	0.006** (0.003)	0.003 (0.003)
	FIRM_PRDT	-0.209*** (0.005)	-0.211*** (0.005)	-0.010*** (0.001)	-0.010*** (0.001)	-0.013*** (0.003)	-0.010*** (0.003)	-0.264*** (0.003)	-0.261*** (0.003)
	FIRM_SIZE	-0.293*** (0.012)	-0.293*** (0.012)	-0.027*** (0.003)	-0.027*** (0.003)	-0.087*** (0.005)	-0.092*** (0.005)	-0.331*** (0.006)	-0.327*** (0.006)
Dummy variables	DESIGN_ENGINEER		-0.088*** (0.023)		-0.020*** (0.006)		0.060*** (0.014)		0.196*** (0.015)
	DESIGNER						-0.084*** (0.015)		0.107*** (0.016)
	Constant	-2.417*** (0.043)	-2.360*** (0.046)	-6.637*** (0.011)	-6.624*** (0.011)	-6.359*** (0.022)	-6.378*** (0.023)	-1.454*** (0.024)	-1.571*** (0.026)
	Observations	4,374	4,374	4,374	4,374	10,293	10,293	10,293	10,293
	VIF	1.60	1.53	1.60	1.53	1.52	1.29	1.52	1.29
	BP (χ^2^) for heterosckedasticity	2039.2***	2004.4***	10982***	10993***	20686***	21117***	1682.5**	1883.9***
	Wooldridge test for autocorrelation	26.511***	22.728***	10.64***	9.0056***	113.5***	103.45***	634.9***	595.64***

Notes

* p < 0.1

** p < 0.05

*** p < 0.01. Standard errors are given in parentheses below the coefficients.

### 4.4 Case studies

#### Apple Inc

**Fig 3:**
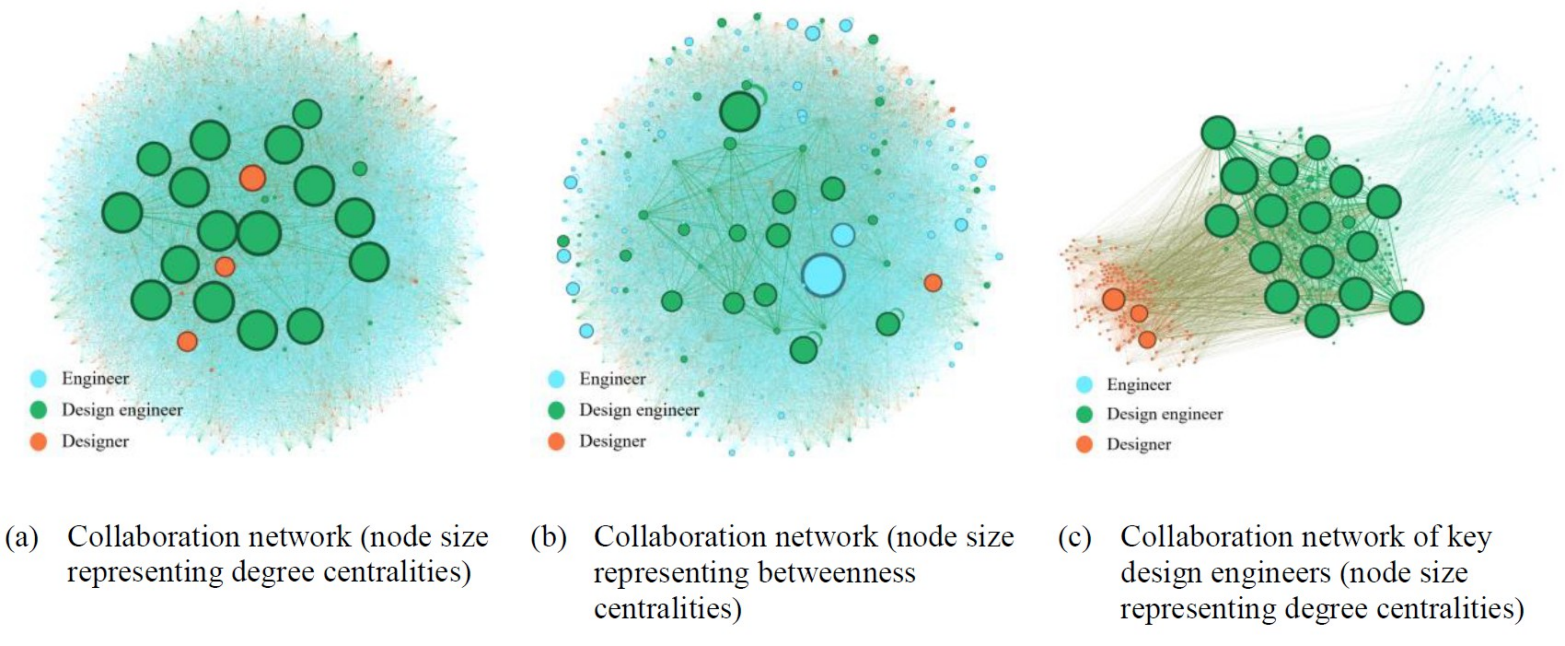
Illustration of Apple’s collaboration networks (period: 2012–2015).

Fig 3 illustrates Apple Inc.’s collaboration networks from 2012 to 2015, with nodes representing inventors and ties denoting co-patenting. Fig 3(A) presents the entire network, with the node size denoting the degree centrality. Within Apple, a few design engineers stand out, with significantly high degree centralities, indicating extensive collaboration. This supports our expectation that design engineers take on the overall coordination of NPD and facilitate cross-functional communication as key collaborators and knowledge sharers within firms. In contrast, engineers tend to have a low degree centrality, reflecting limited collaborative connections.

In Fig 3(B), we observe the same collaboration network as Apple Inc., but the node size represents the betweenness centrality. Design engineers emerged as key figures within the network, as indicated by their notably higher betweenness centrality values than most other engineers. This underscores the pivotal role of design engineers as mediators and brokers in a collaborative landscape. Their ability to facilitate the flow of information and connections between different parts of a network is evident, thereby enhancing collaboration’s overall efficiency and effectiveness.

In Fig 3(C), we focus on a collaborative network centered on Apple’s key design engineers. This subnetwork highlights the pivotal role of design engineers as mediators and brokers between designers and engineers. They play a crucial role in bridging the gap between these two distinct but interdependent realms of innovation—design and technological innovation—to foster a harmonious integration of design aesthetics and technical excellence in product development.

#### Becton, Dickinson and company

**Fig 4:**
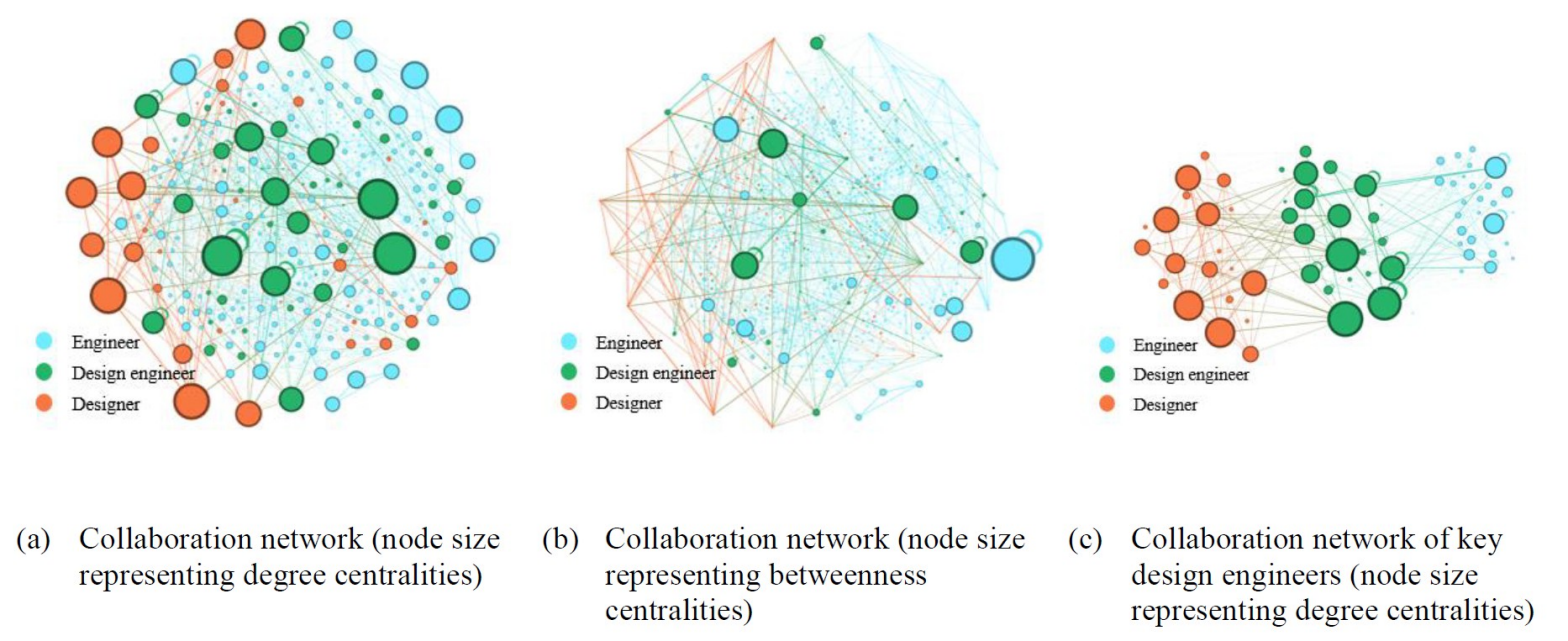
Illustration of Becton Dickinson’s collaboration networks (period: 2012–2015).

Fig 4 illustrates collaborative networks of Becton Dickinson, a medical technology company, from 2012 to 2015. Fig 4(A) provides an overview of the entire collaborative network, with node size indicating degree centrality. Similar to the case of Apple, a select group of design engineers stand out with significantly high degree centralities. Fig 4(B) shifts the focus to betweenness centrality. While a single engineer takes the lead with the highest betweenness centrality, there are notable contributions from design engineers as well. Fig 4(C) underscores the vital role design engineers play as connectors between designers and engineers. Overall, the collaboration network of Becton Dickinson showcases the crucial role of design engineers in knowledge sharing. Their high degree and betweenness centralities, and role as connectors between designers and engineers collectively contribute to the organization’s overall efficiency in product development.

## 5. Discussion and implications

Because inventors are heterogeneous in knowledge, they play different roles in firms’ NPD processes [7]. Design engineers, who possess both design capabilities and technical knowledge, occupy a unique position within this landscape. Their ability to bridge design and engineering makes them pivotal contributors to NPD [14]. To capture the differences between engineers and design engineers in their roles and contributions to NPD, we compared their structural positions in intrafirm knowledge and collaboration networks. Revisiting the research questions, this study examined the differences between engineers and design engineers regarding the combinatorial potential of their knowledge composition and their differences in collaboration patterns. Four sets of network indicators were introduced to identify collaboration and knowledge patterns: degree and betweenness centrality within the collaboration network, and degree and betweenness centrality within the knowledge network. Our analysis revealed several noteworthy observations that have implications for both theory and practice.

The increasing complexity and exponential growth of knowledge have driven inventors to specialize in specific fields, fostering increased combinatorial potential within their respective domains. This specialization is a strategic response to the demand for in-depth expertise in diverse areas of scientific and technological knowledge, aligning with the literature emphasizing the highly specialized nature of expertise in NPD [2, 60]. In contrast, design engineers, with their diverse knowledge spanning design and technology, may face limitations in technical depth. This diversity often translates into specialization in areas (e.g., user interface, user experience) where the convergence of design and technology is essential. While this focused expertise is crucial for their role in integrating design and technology, it can impact their technical depth in comparison to more specialized engineers. Consequently, on average, design engineers possess lower combinatorial potential than engineers, as indicated by their lower degree and betweenness centrality within the firm’s knowledge network. This finding underscores the trade-off between the breadth and depth of expertise and its implications for combinatorial potential [61, 62].

Design engineers are densely connected to other inventors in the collaborative network, although they have knowledge elements that are relatively disconnected in the knowledge network. This nuanced variation in the roles played by design engineers across different types of networks adds to our understanding of their dynamic involvement within an organizational context. These findings align with Wang et al. [17], suggesting that an inventor’s position in the social network is decoupled from the position of their knowledge elements within the knowledge network.

The collaborative cultures of designers and engineers are significantly influenced by decomposability [37]. Engineers, through the breakdown of technology into components, operate within specialized teams, fostering collaboration within their expertise but potentially limiting the degree of collaboration and cross-disciplinary interaction. In contrast, design, characterized as non-decomposable and fundamentally holistic, often leads designers to work independently, resulting in lower degree centralities compared to engineers. In this context, design engineers emerge as pivotal collaborators and knowledge brokers, as indicated by their high degree and betweenness centrality in collaboration networks. With their unique understanding of design aesthetics and technical feasibility, design engineers facilitate cross-functional collaboration and decision-making in the NPD process. They are crucial in resolving conflicts between designers and engineers by mitigating the barriers to effective collaboration [15]. This mediating role highlights the significance of design engineers in bridging the gap between market conceptualization and the production practicality. These findings also align with previous literature highlighting the role of design engineers in overseeing and coordinating projects across the entire NPD process [14] and in mitigating conflicts between designers and engineers [15].

This study makes several significant contributions to new product development and firm innovation. Most previous studies considered a firm’s inventors a simple collection of engineers and relied primarily on inventor information related to utility patents. However, this study breaks new ground by introducing the concept of design engineers as a distinct category of inventors and recognizing their unique roles at the intersection of design and engineering. Additionally, an empirical analysis of knowledge and collaboration networks using patent data offers valuable insights into the roles of engineers and design engineers, enriching our understanding of how these professionals shape and influence innovation ecosystems. The study underscores the pivotal role of design engineers as mediators and facilitators, bridging the gap between the design and engineering disciplines, which has practical implications for firms striving to optimize their innovation processes.

Recognizing the knowledge and collaborative patterns of inventor groups can guide innovation strategies. Firms may leverage engineers’ greater combinatorial opportunities for technological breakthroughs. In contrast, design engineers can be strategically placed to enhance cross-functional communication and decision-making, particularly between the design and engineering teams. Firms may benefit from creating interdisciplinary teams, including engineers and design engineers to capitalize on their complementary strengths. Furthermore, cultivating collaborative environments encouraging engineers to work closely with designers can improve design-engineering skills. Riel et al. [2] noted that a substantial portion of these integrated engineering skills tends to evolve not primarily during initial education but in an engineer’s professional career, especially when they engage in complex interdisciplinary development projects. This approach promotes the development of engineers’ design engineering skills and fosters a culture of cross-disciplinary innovation. Such collaborative environments enhance the potential for breakthrough innovations that seamlessly integrate technological prowess with user-centric design principles. Furthermore, the integration of design and technology is particularly crucial for firms targeting consumers as end-users, such as in the case of consumer goods compared to industrial goods. Companies in these sectors should pay special attention to the role design engineers play in fostering knowledge sharing between designers and engineers, ensuring the integration of design and technology in NPD.

This study also offers insights that can shape education and policies in the broader context of innovation ecosystems. Educational institutions can adapt their design and engineering programs to incorporate more interdisciplinary projects and coursework. This adaptation would help future engineers and design professionals develop the skills necessary for effective collaboration across disciplines. Government and industry policies can play a crucial role in incentivizing collaborative efforts between design and engineering professionals. Funding initiatives that encourage interdisciplinary projects and provide resources for collaborative R&D can contribute to a more integrated and innovative ecosystem. Policymakers can recognize the unique contributions of design engineers in innovation processes. This may involve acknowledging design engineering as a distinct field of expertise and considering policies (e.g., educational and industrial policies) to support its growth.

Our study has some limitations that should be addressed in future research. First, it relies on patent data to determine inventors’ job positions, which might not fully capture their contributions or roles (e.g., project manager) within the firm. Second, our data are limited to US-headquartered public firms, which are relatively large and where job roles tend to be more specialized; therefore, more people need to be involved in product development. The role of design engineers may differ depending on the firm size. Future research could adopt a more comprehensive approach by delving into organization-specific and industry-specific differences in the roles of inventors. Conducting case studies across diverse organizational contexts and industries would allow for an examination of how the unique characteristics of different firms and industries shape the contributions and challenges faced by inventors. This approach could unveil contextual factors influencing the effectiveness of collaborative efforts and help tailor innovation strategies to specific organizational and industrial landscapes.
